# Automatic Coding of Facial Expressions of Pain: Are We There Yet?

**DOI:** 10.1155/2022/6635496

**Published:** 2022-01-11

**Authors:** Stefan Lautenbacher, Teena Hassan, Dominik Seuss, Frederik W. Loy, Jens-Uwe Garbas, Ute Schmid, Miriam Kunz

**Affiliations:** ^1^Physiological Psychology, University of Bamberg, Bamberg 96047, Germany; ^2^Center for Cognitive Interaction Technology CITEC, Bielefeld University, Bielefeld 33619, Germany; ^3^Electronic Imaging Department, Fraunhofer Institute for Integrated Circuits IIS, Erlangen 91058, Germany; ^4^Cognitive Systems, University of Bamberg, Bamberg 96047, Germany; ^5^Digital Sensory Perception, Fraunhofer Institute for Integrated Circuits IIS, Erlangen 91058, Germany; ^6^Medical Psychology and Sociology, University of Augsburg, Augsburg 86159, Germany

## Abstract

**Introduction:**

The experience of pain is regularly accompanied by facial expressions. The gold standard for analyzing these facial expressions is the Facial Action Coding System (FACS), which provides so-called action units (AUs) as parametrical indicators of facial muscular activity. Particular combinations of AUs have appeared to be pain-indicative. The manual coding of AUs is, however, too time- and labor-intensive in clinical practice. New developments in automatic facial expression analysis have promised to enable automatic detection of AUs, which might be used for pain detection.

**Objective:**

Our aim is to compare manual with automatic AU coding of facial expressions of pain.

**Methods:**

FaceReader7 was used for automatic AU detection. We compared the performance of FaceReader7 using videos of 40 participants (20 younger with a mean age of 25.7 years and 20 older with a mean age of 52.1 years) undergoing experimentally induced heat pain to manually coded AUs as gold standard labeling. Percentages of correctly and falsely classified AUs were calculated, and we computed as indicators of congruency, “sensitivity/recall,” “precision,” and “overall agreement (F1).”

**Results:**

The automatic coding of AUs only showed poor to moderate outcomes regarding sensitivity/recall, precision, and F1. The congruency was better for younger compared to older faces and was better for pain-indicative AUs compared to other AUs.

**Conclusion:**

At the moment, automatic analyses of genuine facial expressions of pain may qualify at best as semiautomatic systems, which require further validation by human observers before they can be used to validly assess facial expressions of pain.

## 1. Introduction

For clinical pain assessment, facial responses to pain are of great diagnostic relevance beside the subjective responses, especially for verbally undeveloped or impaired individuals, like young children, children with intellectual disability, or aged individuals with dementia or aphasia [1, 2]. For these individuals, observational pain assessment tools have been developed (e.g., PAIC-15 (Pain Assessment in Impaired Cognition) [3], Doloplus (Behavioral Pain Assessment in the Elderly) [4], and PAINAD (Pain Assessment in Advanced Dementia) [5]), which all include items for assessing facial responses to pain [6].

The very promising diagnostic potency of facial responses to pain has also attracted the interest of several research groups that aim at developing automatic pain recognition systems based on video recordings of facial expressions [7]. So far, the standard assessment of facial expressions of pain is performed by human observers, either by the use of behavioral observation scales (see Herr et al. [8] for a review on pain observation scales) or by the use of fine-grained analyses using the Facial Action Coding System (FACS [9]), which provides so-called action units (AUs) as parametrical indicators of facial muscle activity. Both approaches have their shortcomings. On the one hand, observational scales are still suffering from insufficient reliability/validity and implementation barriers such as the unwillingness of clinical staff to change seasoned routine, the expected training expenditure, and increasing staff requirements [6, 10]. Manual FACS coding, on the other hand, is very time- and labor-intensive given that coding of one minute of video material can take up to two hours and approximately 100 hours of training is needed to achieve FACS certification as a coder [9]. Moreover, both approaches mainly target acute pain (postsurgical, procedural, and acute injuries and diseases) but not chronic pain and do not allow for a constant monitoring of pain as they are based on relatively short observational time windows. Since pain often occurs in episodes or fluctuates in intensity, only constant monitoring would prevent missing critical pain events. These shortcomings limit the clinical usefulness of manual approaches via human observers. Thus, the question arises whether automatic pain detection systems might be a good alternative and whether they are performing better than human observers.

There are mainly two types of systems for automatic analyses of facial pain expression, namely, one-step and two-step approaches. The one-step approaches predict pain or pain intensity based on geometric, textural, and/or temporal features extracted directly from the input image or image sequence. The two-step approaches use or require an intermediate learning stage for describing the facial expression in terms of action units (AUs) [11], making the AU the intermediate result between the videotaped facial expression and the pain estimation. While the one-step approaches correspond to the classical way of learning the target from the input features, the two-step approaches are motivated by the way in which human observers detect and code pain—on the basis of specific facial expression elements as described by AUs [11]. An extensive summary of machine learning methods used so far in automatic pain detection in facial videos can be found in a recent review of Hassan et al. (see especially Table 7 of [7]).

AUs have been studied over many years using manual FACS coding and proven valid as pain-indicative facial behaviors [12]. Because of that, they can be used for gold standard labeling (GSL) in comparison with automatic algorithms. Pain can be inferred from the occurrence of certain AUs and their varying combinations, namely, lowering the brows (AU4, the AU number system has been determined by the developers of the system), cheek raise/lid tightening (AUs6_7), nose wrinkling/raising the upper lip (AUs9_10) and opening of the mouth (AUs25_26_27), and closing of the eyes for longer than half a second [12, 13]. In the present study, we call these AUs “pain-relevant AUs.” The remaining AUs from the FACS are labeled as “nonrelevant AUs.”

Regarding the two-step approaches, specifying the AUs in an intermediate step provides a form of transparency and a high degree of comparability in automatic pain recognition systems and is therefore favored as most promising for the future. Therefore, we also favored two-step approaches for the present study. However, the essential question still is how good the automatic AU detection already is when compared to the GSL by the well-validated manual FACS coding method. For comparison with the GSL, we selected the professional software FaceReader [14, 15] developed by Vicarious Perception Technologies B.V. Amsterdam and distributed by the company Noldus (Noldus Information Technology bv, Wageningen, The Netherlands) because it was the first commercially available, advanced automatic algorithm that allowed detecting single AUs and was the global market leader among AU detection algorithms (AUDA) at the time of the present study. A few previous attempts have been made to investigate the agreement between manual FACS coding and FaceReader, showing satisfactory agreement [15, 16]. However, these findings were based on the analyses of posed facial expressions of other emotions than pain by young actors and thus on datasets showing artificially ideal conditions with highly controlled recordings of strong facial expressions. The unique feature of our study was the use of real facial expressions of pain in an age group commonly underrepresented in the study of facial expressions (i.e., middle adult-aged individuals), which forms however the majority of the pain patients [17]. Thus, including older individuals in such automatic facial expression analyses is crucial for the clinical usefulness of such systems. With regard to using facial responses to experimentally induced compared to clinical pain, previous studies have shown that the AU patterns of pain are very similar for experimental and clinical pain [12, 13].

Consequently, the question remains of how well the automatic detection of AUs performs on more real facial expression data, namely, on genuine facial responses to experimentally induced pain in individuals of different age ranges. Prior research concerning the influence of age on facial communication of pain has indeed shown that age does not severely impact how pain is facially expressed [18]; however, age influences how observers rate the facial responses [19–21]. Thus, we investigated the automatic detection of facial responses to pain by FaceReader in a naturalistic age range also relevant for pain patients [17] and compared the automatic detection between younger and older (middle-aged) individuals. The present study is one of the first that challenges a state-of-the-art AUDA with a collection of real pain expressions in middle adult-aged individuals.

The main aim of our study was to compare manual with automatic AU coding of facial expressions of pain and to answer the questions: (i) whether the AUs relevant for pain (“pain-relevant AUs”) are better detected than other AUs (“nonrelevant AUs”) and (ii) whether the age of the observed individual matters for the quality of detection. Given that the systems have been mostly trained with facial expressions of young adults [7], we expect that the FaceReader has only moderate to low agreement with the GSL of the manual coding, especially when analyzing facial responses to pain in older individuals.

## 2. Materials and Methods

Our study is an exploratory study, using an observational, cross-sectional study design.

### 2.1. Dataset

#### 2.1.1. Participants

The sample was composed of 40 healthy adults. They were divided into two subsamples: 20 younger (20–39 years; mean age = 25.7 ± 4.0; 65% female) and 20 older participants (40–65 years, mean age: 52.1 ± 7.0; 65% female). This is a subset of a full dataset of 126 participants taken from an earlier study [22], chosen based on (a) the availability of high-quality frontal view videos (*N*  = 9 excluded), (b) no glasses or hair masking any part of the face (*N*  = 17 excluded), (c) the stable occurrence of facial responses to the experimental pain protocol (*N*  = 44 excluded), (d) equal proportion of males and females in each age group (*N*  = 4 (younger males) excluded), and (e) equal numbers of younger and middle-aged participants (*N*  = 12 (younger individuals excluded). These criteria resulted in a small gender imbalance (7 males vs. 13 females in each age group). The rational for selecting just these two age groups is described in the following sentences. Most of the computer algorithms were commonly trained on student populations, and thus, our younger group was typical for most tests published [23]. However, individuals of this age rarely suffer from clinical pain. Thus, we selected an older group with an average age, which represents an age range typical for patients suffering from chronic pain. The required sample size for an exploratory study is a minimum of 15 subjects per group [24]. With a minimal group size of 20 subjects (young and old age group), our sample size seems sufficient.

More details on the original sample (e.g., recruitment and inclusion/exclusion criteria) and the study protocol can be found in Karmann et al. [22].

#### 2.1.2. Procedure

In short, participants were recruited via advertisement in local newspapers (Bamberg, Germany). All participants provided written informed consent and received monetary compensation. Each participant was video recorded during thermal stimulation applied on the outer part of the left lower leg by a Peltier-based contact stimulation device (Medoc, TSA-2001, Ramat Yishai, Israel) with a 30 mm ∗ 30 mm contact thermode. During stimulation, participants were seated in a comfortable chair facing a computer screen and were instructed to sit still, keep their focus on the computer screen, and refrain from talking during the stimulation in order to gain a good frontal view of the face. The thermal stimulation was tailored to the individual pain threshold (method of adjustment). Thermal stimulation was composed of 10 painful (+3°C above the pain threshold) and 10 nonpainful (−3°C below the pain threshold) phasic stimuli (trapezoid form; 5 s plateau; rate of rise: 4°C/s; baseline temperature: 38°C; interstimulus intervals of 10–15 s) which were applied in a random order. The stimulation intensities of plus/minus 3°C in relation to the individual pain threshold are based on previous studies where we could show stable nonpainful and painful experiences at these intensities [25].

#### 2.1.3. Materials

In this paper, we only focus on the facial responses to the painful stimuli, given that the nonpainful stimuli were accompanied by very little facial responses (bottom effect). Thus, the video dataset used in this paper contains 10 (painful stimuli) × 40 (participants) = 400 video sequences, each lasting for 5 seconds (duration of the painful stimuli at plateau), with a frame rate of 30 frames per second.

### 2.2. Manually Coded FACS Analysis (GSL)

Facial expressions were manually coded from the video recordings using the Facial Action Coding System [9], which is based on anatomical analysis of facial movements and distinguishes 44 different action units (AUs) produced by single muscles or combinations of muscles. Two certified FACS coders (qualified by passing an examination given by the developers of the system) identified the frequency and the intensity (5-point scale) of the different AUs. Inter-rater reliability was calculated using the Ekman–Friesen formula [9] and reached 0.87, thus exceeding the criterion for FACS certification (>0.70) and indicating good reliability. A software designed for the analysis of observational data (the Observer Video-Pro; Noldus Information Technology) was used to segment the videos and to enter the FACS codes into a time-related database. Manual FACS coding requires 15 to 20 times the length of video raw material for AU detection.

### 2.3. Automatic FaceReader Analysis

The software FaceReader (version 7, Noldus, Wageningen, The Netherlands) was used to conduct the automatic FACS analysis. This analysis was carried out on a Lenovo ThinkPad (15.6 FHD, 1920 × 1080) which met the system requirements as described in the FaceReader manual [15]. Image resolution of the videos was 1024 × 576 pixels.

We used the basic default FaceReader settings for the analysis of the video sequences. Thus, the general default face model (having been trained on a wide variety of images) was chosen; the sample rate was set to every frame. The classification of the basic emotion expressions was discarded, and the AU classification was activated (the default specification allows for a binary decision on whether an AU is coded as present or not). FaceReader is capable of classifying 20 AUs (out of the 44 AUs defined in the manual FACS manual). These AUs are listed in Table 1. For further analyses, we used the binary information for each AU (present − not present).

### 2.4. Indices of Comparison

In the manual FACS coding of facial expressions of pain, the agreement between FACS coders is analyzed for each pain event using the agreement index [9]. To approach the comparison between manual FACS coding and the FaceReader analysis in a similar way, we extracted for each pain event which AUs were coded from both the manual FACS coding and FaceReader. Thus, we only compared the congruency regarding the types of AUs being detected manually and automatically, regardless of the congruency in AU intensity or congruency in the exact frame-by-frame occurrence of the AUs. Only the 20 AUs that are annotated by FaceReader are analyzed (see Table 1). Although the manual FACS coding included all 44 AUs, there was not a great loss due to the restriction to the 20 AUs, given that these covered the most frequently occurring AUs. Indeed, whereas the average frequency value of the 20 included AUs reached a value of 110 (see Table 1, first column), the average frequency value of the omitted AUs was only 8.

Following previous approaches comparing FaceReader performance to manual FACS coding (GSL) [14, 16], the congruency between the two was calculated using (i) recall/sensitivity, (ii) precision, and (iii) F1 which were computed for each of the 20 AUs separately. Recall/sensitivity denotes the ratio of manually coded AUs that were also detected by FaceReader. Precision denotes the ratio of how often the FaceReader AU classification is consistent with the manual FACS coding. F1 summarizes the trade-off between recall/sensitivity and precision via the formula [26]:

(1) $$\begin{array}{c} 2*\frac{\left(\text{Precision}*\text{ Recall}\right)}{\left(\text{Precision }+\text{ Recall}\right)}. \end{array}$$

The F1-index uses the harmonic mean, to represent this trade-off. The 2 represents the number of compared indices (namely: “recall” and “precision”).

Calculating all three indexes allows a direct comparison of our findings and those of previous studies [9, 14, 16].

### 2.5. Statistical Procedure

In order to investigate whether the three indexes might differ depending on whether an AU was pain-relevant or not, we compared the indexes between pain-relevant AUs [8, 9] and AUs that have not shown a strong linkage to pain using Wilcoxon tests. Moreover, we also compared indices between young and older faces using Mann–Whitney U tests. All analyses were conducted using SPSS 26 (IBM SPSS Statistics 26 Documentation).

## 3. Results

### 3.1. Overall Performance of FaceReader

Table 1 gives an overview of the congruency between manual FACS coding and FaceReader separately for the 20 AUs. The first column “Present” refers to the frequency with that an AU was manually FACS coded (GSL) across all 400 video segments used in the present study or was automatically detected by FaceReader, respectively.

  Recall/sensitivity: the recall value of 0.47 for AU1 indicates, for example, that only 47% of the manually coded AU1 was also classified as such by FaceReader (see Table 1). Highest recall values were found for AU4 (brow lowering) and AU43 (closing of the eyes), with approximately 2/3 of these facial responses being detected by FaceReader. Across all 20 AUs, the recall was quite low, with only 1/3 of the AUs being detected by FaceReader.
  Precision: in the case of AU1, the FaceReader classification was consistent with the manual FACS coding in only 39% of the time to give an example (see Table 1). Highest precision values were found for the degree of mouth opening (AU25, AU26). Across all 20 AUs, the precision was quite low, with the FaceReader classification being consistent with the manual FACS coding only 43% of the time.
  F1: the integration of measures of precision and recall shows an acceptable agreement between FaceReader and manual coding only for AU4 (see Table 1). All other AUs show only low agreement rates (F1).

### 3.2. FaceReader Performance: Pain-Relevant versus Nonrelevant AUs

**Figure 1:**
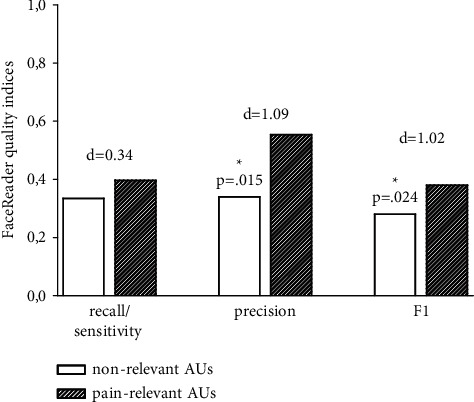
FaceReader7 performance in dependence of whether AU is pain-relevant or nonrelevant. Nonrelevant AUs: AU1, AU2, AU5, AU12, AU14, AU15, AU17, AU18, AU20, AU23, AU24. Pain-relevant AUs: AU4, AU6, AU7, AU9, AU10, AU25, AU26, AU27, and AU43. ^*∗*^*p* < 0.05; *d* = *effect size, Cohen*'*s d*.

We divided the AUs into those AUs that have been found to be relevant for pain (AU4, AU6, AU7, AU9, AU10, AU25, AU26, AU27, and AU43) and those that do not show such a strong connection with pain (AU1, AU2, AU5, AU12, AU14, AU15, AU17, AU18, AU20, AU23, and AU24) [12, 13]. When computing the three quality indices separately for pain-relevant and nonrelevant AUs (see Figure 1), we found that FaceReader showed better detection for the pain-relevant AUs. Using Wilcoxon test for nonparametric comparisons revealed that this difference reached significance in the case of precision values (*p*=0.026) as well as for F1 (*p*=0.045). These differences were of large effect size.

### 3.3. FaceReader Performance: Younger versus Older Faces

**Figure 2:**
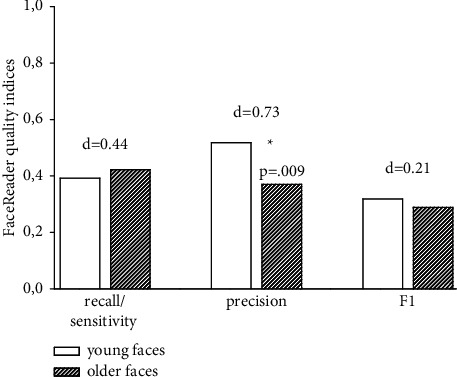
FaceReader7 performance in dependence of whether AU detection was performed in younger (20–39 years) or older (40–60 years) individuals. ∗*p* < 0.05; *d* = *effect size, Cohen*'*s d*.

We also computed the three quality indices separately for the videos showing facial responses in younger and in older individuals. As can be seen in Figure 2, FaceReader showed slightly better AU detection in younger compared to older individuals. Using Mann–Whitney U test for nonparametric comparisons revealed that this difference reached significance in case of precision values (*p*=0.022). This significant difference was of medium effect size.

## 4. Discussion

Over the last decades, manual FACS coding of facial expressions of pain has proven to be a reliable and valid assessment method across a great variety of studies [1, 12] and surely qualifies for gold standard labeling (GSL) the facial responses to pain. Compared to this GSL, the automatic system FaceReader for recognizing facial expressions appears fairly disappointing, considering the low congruency between the AU estimates of the two approaches (manual FACS coding vs. FaceReader). When computing congruency values for each single AU, recall/sensitivity of FaceReader varied around 36%. It is noteworthy that two pain-relevant AUs, namely, AU4 (brow lowerer) and AU43 (closing of the eyes >0.5 seconds), showed good recall/sensitivity with 63% and 71%, respectively. Moderately good quality could be obtained when computing the ratio of how often FaceReader is correct when classifying an AU as being present (precision). These estimates could reach percentages of around 60–70%, especially for those AUs, which are relevant for pain (AU4 = 67%; AU6 = 67%; AU10 = 54%; AU25 = 74%; and AU26 = 73%). Thus, some of the pain-indicative AUs showed at least somewhat promising outcomes. However, it also has to be mentioned that one of the most prominent facial responses during pain, namely, AU7, only reached low recall/sensitivity (36%) and precision (44%) scores. Moreover, the trade-off between recall and precision, namely, the F1 value, also showed weak agreement between FaceReader and manual FACS coding (varying around 32%). This was also the case for most of the pain-indicative AUs, with two exceptions: AU4 (65%) and AU25 (57%).

When comparing our findings with previous attempts comparing manual FACS coding with FaceReader performance, it becomes apparent that previous studies have rendered much more favorable outcomes [14, 16]. In these previous studies, the FaceReader performance was compared to manual FACS coding using recordings of posed facial expressions of emotions by mostly young actors in ideal recording conditions (e.g., good lighting and stable frontal view of the face). Here, the values for recall, precision, and F1 for AU detection reached scores of around 70% (recall: around 0.75%; precision: around 0.65%; F1: around 0.65%) [14, 16]. There are no studies comparing manual FACS coding and FaceReader performance on data showing genuine facial expressions of emotions or of pain. A recent study [27], however, compared manual and automatic AU detection on videos showing genuine facial expressions of pain in older individuals using other automatic facial expression software, namely, OpenFace [28] and Affdex SDK [29]. Interestingly, the findings of that study are very comparable to our outcomes, namely, that the congruency between manual and automatic AU detection was only poor (0.3–0.4%). Moreover, another recent study also compared manual and automatic AU detection in videos showing genuine facial expressions of pain and disgust using the software Computer Expression Recognition Toolbox (CERT [29]) and also found very poor agreement [30]. Thus, automatic AU coding seems accurate only when videos are captured under artificially ideal conditions and when facial expressions are posed by young actors [31]. If facial expressions are, however, recorded in genuine pain or emotion eliciting situations, the congruency drops markedly. Thus, we think that it was a necessary further step to introduce analyses of real pain faces, not only in younger individuals but also in people of an age typical for chronic pain sufferers.

### 4.1. Comparing the Congruency between Pain-Relevant and Pain Nonrelevant AUs

All three indices of congruency showed better outcomes for those AUs that have been found to be relevant for pain [12]. To be clear, these were also the AUs with higher frequency of occurrence compared to the other AUs, which might have prevented floor effects. For pain-relevant AUs, FaceReader performed best regarding the quality index “precision.” In other words, whenever FaceReader detected a pain-relevant AU, this detection was mostly consistent with the manual FACS coding (in around 60% of the cases). However, FaceReader also missed many of the pain-relevant AUs, given the low values for recall/sensitivity. Thus, FaceReader appeared to be largely precise in the detection of pain-relevant AUs (moderate hit rate) at the expense of missing many of those AUs (high miss rate). From the perspective of pain diagnosticians, FaceReader appeared to be a device favoring cautious decisions, which both avoids false alarms and tends toward false-negative decisions. This bias is not ideal for a screening device—the automatic pain diagnosis systems will likely be used as such in the future—because necessary pain alarms allowing the nurse or physician to validate pain are not activated. Thus, just the opposite bias would be favorable for the likely implementation.

### 4.2. Comparing Congruency between Younger and Older Faces

We found that FaceReader was more often consistent with manually FACS coding when detecting an AU to be present (precision) in younger faces compared to older faces. Given that automatic facial expression software is mostly trained using the faces of young individuals, it is not surprising that the performance was slightly better for younger faces, given that skin color and texture do vary depending on age [32, 33]. We even expected greater differences in automatic AU detection between the age groups. It is possible that the age gap between the two groups was not large enough and that only faces of very old individuals, which lack any similarity with the young individuals commonly used for AUDA training, might lead to a clear reduction in AUDA performance. Regardless, it is promising that only the quality index “precision” showed a slight age bias in the present study, whereas the other indices were not affected. For the final validation of AUDAs, databases are however needed, which contain videos of pained faces over the full age range. Ideally, such databases would also include other relevant features for pain expression like gender, ethnicity, and psychological state, which would make the collaboration of many centers necessary.

Overall, given the low to moderate congruency values, FaceReader7 in its present form may qualify at best as semiautomatic system, which still urgently requires a human observer for validation. In reaching this standard, it is in best company with other comparable automatic systems (e.g., OpenFace and Affdex) [31]. Even if these systems can only be used for semiautomatic AU detection, this could still present substantial assistance for manual FACS coding. If the suggestions of the automatic systems help the human observer to speed up the coding, the disadvantage of slowness of manual FACS coding might partially be overcome. However, this requires appropriate interfaces between human and machine, which allow for validation of the suggestions provided by the automatic systems.

Valid and reliable detection of AUs related to pain is the first critical step for the transparent automatic pain recognition. For complete pain diagnostics or pain monitoring in clinical contexts, the next necessary step is to infer from the occurrence of certain AUs the existence of pain. To again highlight this feature of two-step approaches, all AUDAs, as also FaceReader, provide as an intermediate result AU pattern, which have to be subjected to a second step, namely, the AU pattern interpretation. For note, this second step cannot be accomplished by considering single AUs alone but by also considering combinations of AUs typical for pain. We could demonstrate that the facial expression of pain consistently occurs in clusters of interindividually varying combinations of AUs in both clinical and experimental pains [34]. Only by considering these combinations or even better by using individualized learning algorithms to detect the individual's expression cluster, will it be possible to use automatic facial expression systems for clinical pain diagnostics.

A few words may be warranted to describe the lines of development of automatic pain diagnostic devices based on the facial expression of pain, which FaceReader may be part of. Given that, at the moment, constant automatic pain monitoring has nowhere routinely been implemented, the first steps may be application in the recovery rooms in the acute postoperative phase, with monitoring phases from minutes to a few hours. This means that at the beginning only controlled professional environments for mainly bedridden individuals with very likely pain episodes, allowing the implementation of appropriate video technique and pain treating staff close by, will qualify. The development of mobile devices for constant monitoring seems to be the future. Mobile devices to monitor only short pain episodes on the basis of smartphones may already be available in the nearer future. However, first technical and methodological solutions still show many shortcomings [35]. Altogether, automatic pain assessment based on face videos will have a great future given that much personal, technical, and financial effort will be invested because it finally allows studying pain in natural contexts with all its biopsychosocial influences. These biopsychosocial influences may range from gender influences [36], to influences due to culture, ethnicity [37], learning [38], personality [39], education, etc. [40], which should be considered in the future studies together with facial expressions.

Limitations of the present study: we have to acknowledge some limitations to our exploratory study. First, the proportion of women and men in the video sample was not perfectly balanced which prevented us from conducting gender comparisons. Moreover, our sample was very homogenous as regards the cultural and ethnic background, with all individuals being Caucasians from Germany. Samples that are more diverse as regards age, ethnicity, culture, and physical and psychological states are needed in order to train and validate automatic systems. Although we believe our sample size to be sufficient for an exploratory study, future studies using larger sample sizes should be conducted to also improve the diversity issue just discussed and thereby increase the external validity of the findings.

## 5. Conclusions

The present study showed that at the moment, automatic analyses of genuine facial expressions of pain (using FaceReader) may qualify at best as semiautomatic systems, which require further validation by human observers before they can be used to validly assess facial expressions of pain. We have to acknowledge, however, that our approach is one of the first attempts to test FaceReader on facial expressions during pain and further in-depth analyses (a) using larger sample sizes, (b) applying a larger range of pain stimuli, and (c) considering the intensities of the manually coded AUs are needed to better understand the strength and weaknesses of the automatic system.

## Figures and Tables

**Table 1:** Congruency indices between manual and automatic AU coding.

Action unit	Present		Recall/sensitivity	Precision	F1
	GSL	FaceReader			
1—inner brow raiser	98	94	0.47	0.39	0.43
2—outer brow raiser	92	30	0.32	0.48	0.38
4—brow lowerer	393	300	**0.63**	**0.67**	**0.65**
5—upper lid raiser	16	23	0.32	0.12	0.17
6—cheek raiser	174	31	0.15	**0.67**	0.25
7—lid tightener	252	154	0.36	0.44	0.40
9—nose wrinkler	74	44	0.32	0.45	0.38
10—upper lip raiser	151	40	0.27	**0.54**	0.36
12—lip corner puller	50	47	0.40	0.28	0.34
14—dimpler	151	38	0.15	**0.59**	0.25
15—lip corner depressor	33	69	**0.55**	0.19	0.29
17—chin raiser	102	138	**0.54**	0.29	0.38
18—lip pucker	66	22	0.15	0.46	0.23
20—lip pucker	18	4	0.11	**0.50**	0.18
23—lip tightener	83	75	0.34	0.28	0.30
24—lip pressor	39	61	0.33	0.16	0.21
25—lips part	195	99	0.46	**0.74**	**0.57**
26—jaw drop	161	50	0.27	**0.73**	0.40
27—mouth stretch	1	1	0.00	0.00	0.00
43—closing of the eyes	56	136	**0.71**	0.19	0.30
**Average**	110	73	0.36	0.43	0.32

GSL: gold standard labeling (manual FACS coded data); Present: the number of times an AU was manually FACS coded or coded by FaceReader, respectively; Recall/sensitivity: ratio of manually coded AUs that were correctly detected by FaceReader; Precision: ratio of how often FaceReader is correct when classifying an AU as present; F1: trade-off between recall/sensitivity and precision via the formula: 2*∗*(Precision*∗*Recall)/(Precision+Recall). Note: higher values are marked in bold.

## Data Availability

All anonymized statistical data used and/or analyzed during the present study are available from the corresponding author on reasonable request, but the original video files cannot be shared because they are protected by the prevailing data protection declaration.
